# Drug-loaded PEG-PLGA nanoparticles for cancer treatment

**DOI:** 10.3389/fphar.2022.990505

**Published:** 2022-08-19

**Authors:** Dan Zhang, Lin Liu, Jian Wang, Hong Zhang, Zhuo Zhang, Gang Xing, Xuan Wang, Minghua Liu

**Affiliations:** ^1^ Department of Pharmacology, School of Pharmacy, Southwest Medical University, Luzhou, China; ^2^ Pharmaceutical Department of Traditional Chinese Medicine, School of Pharmacy, Southwest Medical University, Luzhou, China; ^3^ Department of Gastroenterology, The Affiliated Hospital of Southwest Medical University, Luzhou, China

**Keywords:** PEG, PLGA, nanoparticles, antitumor therapy, targeted modification, drug delivery

## Abstract

Nanoparticles based on single-component synthetic polymers, such as poly (lactic acid-co-glycolic acid) (PLGA), have been extensively studied for antitumor drug delivery and adjuvant therapy due to their ability to encapsulate and release drugs, as well as passively target tumors. Amphiphilic block co-polymers, such as polyethylene glycol (PEG)-PLGA, have also been used to prepare multifunctional nanodrug delivery systems with prolonged circulation time and greater bioavailability that can encapsulate a wider variety of drugs, including small molecules, gene-targeting drugs, traditional Chinese medicine (TCM) and multi-target enzyme inhibitors, enhancing their antitumor effect and safety. In addition, the surface of PEG-PLGA nanoparticles has been modified with various ligands to achieve active targeting and selective accumulation of antitumor drugs in tumor cells. Modification with two ligands has also been applied with good antitumor effects, while the use of imaging agents and pH-responsive or magnetic materials has paved the way for the application of such nanoparticles in clinical diagnosis. In this work, we provide an overview of the synthesis and application of PEG-PLGA nanoparticles in cancer treatment and we discuss the recent advances in ligand modification for active tumor targeting.

## 1 Introduction

Tumor malignancies are the second leading cause of death worldwide and their treatment remains expensive and complex (Wiśniewski et al., 2020). Tumor drug therapies mainly include treatment with cytotoxic small molecules, drugs targeting genes or other molecules, as well as active substances of traditional Chinese medicine (TCM). Although more than half of the 170 drugs used for cancer treatment target specific molecules (Levêque and Becker, 2019), chemotherapy and adjuvant TCM treatment are also widely used in clinical practice due to their relatively low price and good therapeutic effect. However, current therapeutic agents suffer from low bioavailability, rapid elimination *in vivo*, high toxicity to normal host cells, and low retention at the tumor site. Peptides used in anti-tumor therapy have the advantages of good membrane permeability and high specificity, but their disadvantages such as high cost, short circulating half-life and rapid clearance *in vivo* limit their wide clinical application (Firer and Gellerman, 2012; Ciobanasu, 2021). The new generation of nanocarrier materials MOFs (metal organic frameworks) has certain potential in disease diagnosis and drug targeted delivery (Karami et al., 2021). However, problems such as the high cost of MOFs synthesis, premature drug release caused by rapid metabolism *in vivo* or clearance by the immune system, carrier stability, and lack of biocompatibility studies still indicate that clinical application of MOFs needs more exploration (Cai et al., 2020).

Nanoparticles (NPs) have been identified as ideal carriers for various types of drugs due to their biodegradability, biocompatibility, storage stability, and easy surface modification (Mitchell et al., 2021). In addition, their particle size and unique surface properties make them suitable for passive targeting of solid tumors: due to the enhanced permeability and retention (EPR) effect, NPs can be retained within the rich blood vessels and on the extensive vascular surfaces in the tumor tissue, where there is no lymphatic reflux to clear them away. For instance, NPs based on the US Food and Drug Administration (FDA)-approved poly (lactic acid-co-glycolic acid) (PLGA) have been widely used to encapsulate almost all types of antitumor drugs, offering good biodegradability, minimal systemic toxicity, and high bioavailability (Khan et al., 2016a). However, their application is limited because intravenously administered PLGA NPs are easily opsonized and rapidly cleared by the reticular endothelial system (Noori Koopaei et al., 2014).

In order to improve the properties of PLGA NPs and achieve long-term therapeutic effects, polyethylene glycol (PEG) has been conjugated with PLGA to construct a new type of amphiphilic block co-polymer nanoplatform, PEG-PLGA NPs. Compared with unPEGylated PLGA nanoparticles, PEGylated PLGA nanoparticles showed a characteristic improvement in the symptoms of multiple sclerosis in mice (Li P. Y. et al., 2021), indicating their potential to improve immune tolerance. Pegylated lipid-PLGA hybrid NPs can significantly reduce the fusion phenomenon of nanoparticles during storage, and further improve the internalization of cell uptake experiments while improving stability (Hu et al., 2015). In studies of PLGA-based magnetic nanoparticles, PEGylation reduces neurotoxicity and improves the stability of the loaded therapeutic DNA in primary hippocampal neurons (Cui et al., 2019). PEGylation also improves the biocompatibility of PLGA-based contrast agent composite nanomaterials to some extent (Xu et al., 2015). The novel preparation showed improved drug encapsulation efficiency and controlled release, especially of chemotherapeutic drugs, active TCM substances, and gene-targeting drugs. In addition, PEG-PLGA NPs exhibited high stability, good bioavailability, and enhanced passive targeting ability by the EPR effect, which promoted the targeted accumulation of the drug at the tumor site and improved its safety.

The surface of PEG-PLGA NPs has also been modified with various ligands, such as glycyrrhetinic acid, chondroitin sulfate, alendronate, polyethylenimine, iRGD (the arginine-glycine-aspartate peptide), and estradiol, in order to allow the NPs to target tumors not only passively but also actively. Extensive studies on the mechanism of highly invasive and metastatic tumors have revealed a large number of abnormally expressed proteins, such as cell adhesion molecules, that can serve as new targets for PEG-PLGA NPs. Cell adhesion molecules are a general class of cell surface transmembrane proteins that mediate cell-cell and cell-extracellular matrix adhesion, especially in tumors. The epithelial cell adhesion molecule (EpCAM) is highly expressed in tumors and it helps regulate the epithelial-mesenchymal transition, giving it a key role in the invasion and metastasis of tumor cells; this molecule can bind specifically to EpCAM aptamer on NPs (Fagotto and Aslemarz, 2020). CD44 is also upregulated in several tumor cell types, and it serves as a marker of cancer stem cells; it can bind specifically to hyaluronic acid on NPs (Chen et al., 2018). The arginine-glycine-aspartate (iRGD) peptide on NPs binds with high affinity to the integrin receptor, which is abundantly expressed on certain tumor types; the peptide can then be internalized, taking the NP and its drug cargo inside the target cells (Davoodi and Shafiee, 2022). The folate receptor is overexpressed in certain tumor types such as ovarian cancer and non-small cell lung cancer, and this has been exploited in several appraoches to develop high-affinity folates for targeted cancer treatment (Ledermann et al., 2015), as well as folate conjugates for chemotherapy, photothermal therapy, and diagnostic imaging (Li et al., 2015; Liu et al., 2018). Another study showed that biotin, a safe water-soluble vitamin, can bind strongly to biotin receptors and the surface of pharmaceutical preparations, showing great potential as an active targeting strategy for cancer treatment (Wang et al., 2020). Furthermore, the great demand of tumor cells for iron leads them to overexpress transferrin receptors on their surface, which might provide another strategy for targeted therapy (Luck and Mason, 2013).

**FIGURE 1:**
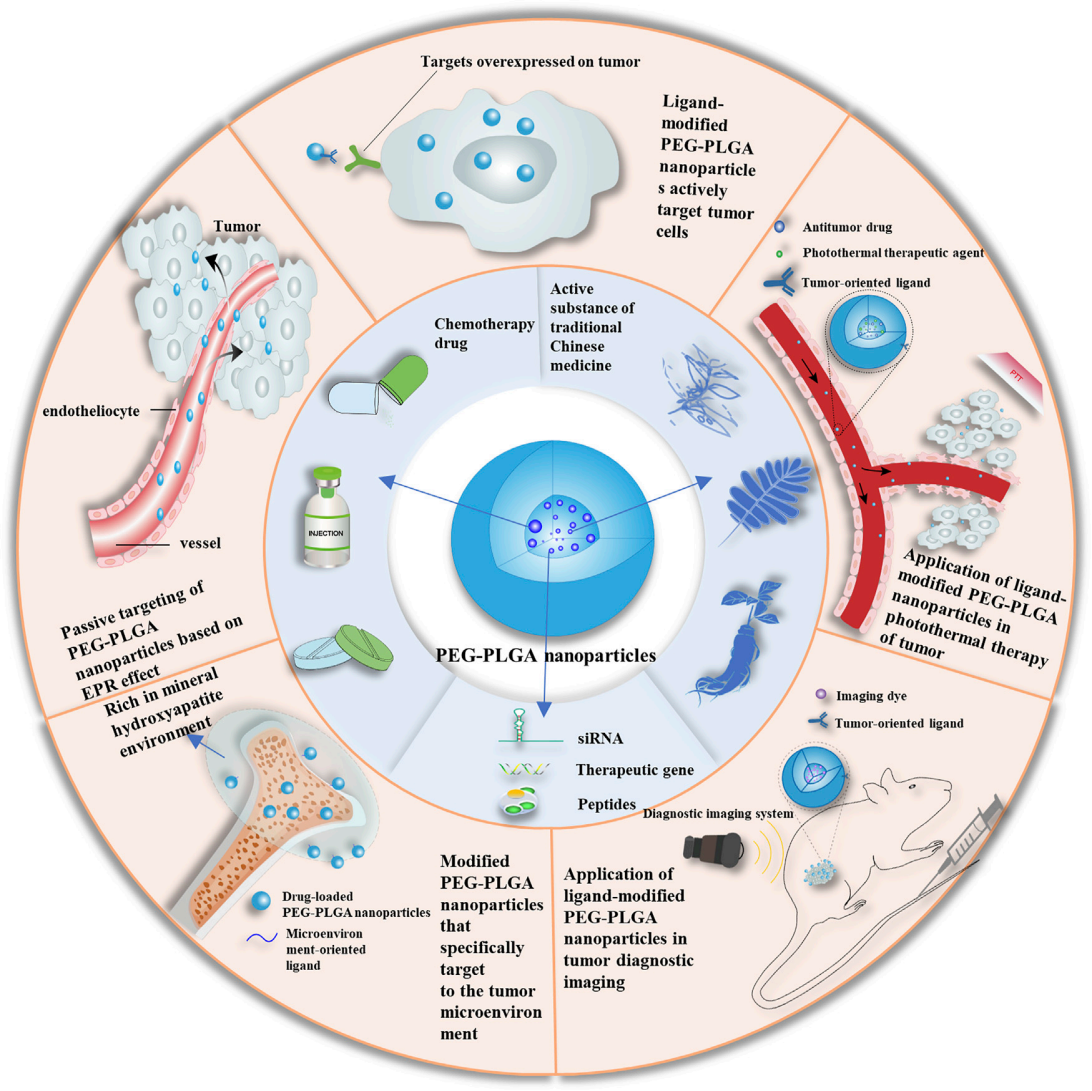
Schematic of applications of PEG-PLGA nanoparticles. EPR, enhanced permeability and retention enhanced permeability and retention; PEG, polyethylene glycol; PLGA, poly (lactic acid-co-glycolic acid); siRNA, short interfering RNA.

In this review, we discuss the formulation principles and properties of PEG-PLGA NPs and summarize the recent advances in their modification and application as drug delivery systems for targeted cancer treatment (Figure 1). The synthesis provided here may guide the development of new antitumor formulations with improved *in vivo* pharmacokinetics, enhanced passive and active targeting, as well as high drug efficiency for effective precision medicine.

## 2 PEG-PLGA NPs

### 2.1 Origin of drug-loaded PEG-PLGA NPs

**FIGURE 2:**
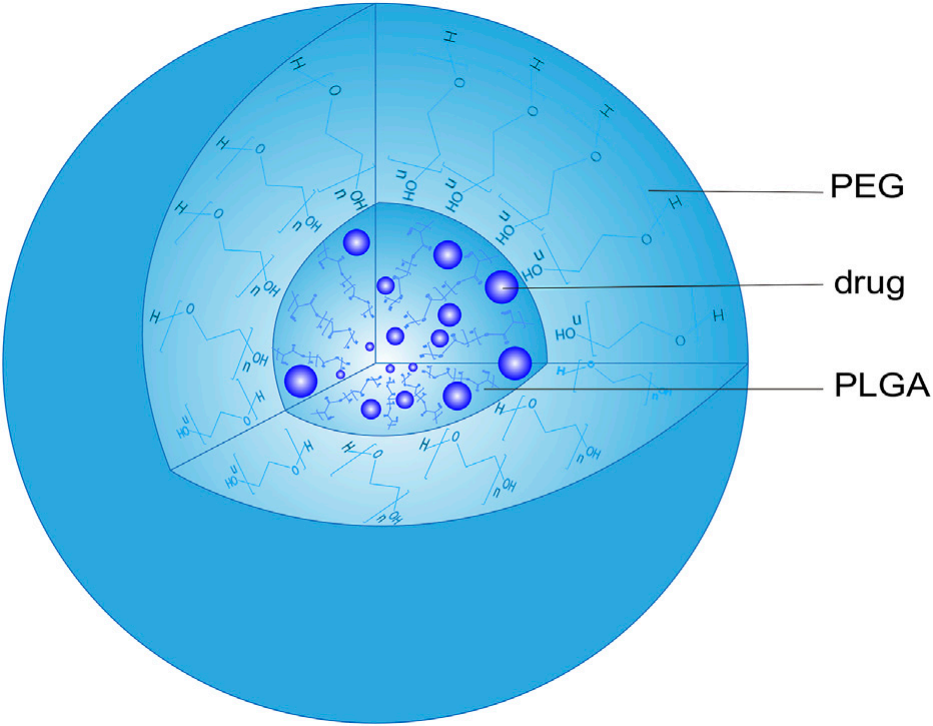
Structure of a PEG-PLGA nanoparticle. PEG, polyethylene glycol; PLGA, poly (lactic acid-co-glycolic acid).

Due to its great biocompatibility and biodegradability, PLGA has been widely used in the preparation of NPs (Lakkireddy and Bazile, 2016). Although PLGA NPs are good carriers for hydrophilic and hydrophobic drugs, their application is limited due to protein opsonization and rapid clearance by the reticuloendothelial system. Adagen was the first PEGylated protein drug approved by the FDA as a treatment for severe combined immunodeficiency (Suk et al., 2016). When PEG covalently binds to the drug surface, it will block antigen determinants to affect antigen-antibody binding to inhibit the immunoreaction. The immunogenicity of ricin against anti-ricin serum can be reduced through PEG modification, which covers epitopes and receptors involved in immune recognition (Hu et al., 2002). PEGylating a genetically engineered form of alginate lyase significantly reduced its ability to be recognized by antibodies from New Zealand rabbits and humans (Lamppa et al., 2011). Similarly, PEGylating porcine follicle-stimulating hormone protected the hormone from immune recognition (Uchiyama et al., 2010). The PEG surface barrier can also protect the drug from enzymatic degradation and rapid elimination by the kidney, prolonging the half-life of the drug *in vivo*. PEGylating recombinant human interleukin-11 (IL-11) not only enhanced its pharmacological activity, but also prolonged its retention time in plasma by reducing the liver and kidney clearance of IL-11 (Takagi et al., 2007) (Menkhorst et al., 2009). Modifying PLGA NPs with PEG improves their surface hydrophilicity and prolongs circulation time (Noori Koopaei et al., 2014), giving them substantial promise as drug carriers (Haggag et al., 2018; Dunn et al., 2019). These NPs consist of a PEG shell and a PLGA core that can effectively encapsulate hydrophilic and hydrophobic drugs (Figure 2).

### 2.2 Preparation of PEG-PLGA NPs

Nanoprecipitation and double emulsion-solvent evaporation are the two main methods for the synthesis of PEG-PLGA NPs; they take advantage of the self-assembly of PEG and PLGA at a specific ratio and temperature. Below we describe only the basic synthetic routes and characteristics of the two methods, since we cannot cover the large variability in excipients, component proportions and reaction conditions that have been explored.

#### 2.2.1 Nanoprecipitation

Nanoprecipitation is a simple preparation method for NPs with narrow particle size distribution that requires low amounts of surfactant, generates few toxic products, and can be performed on a large scale. For the preparation of PEG-PLGA NPs, PEG and PLGA are dissolved in a suitable solvent, mainly acetone, and then added to an aqueous phase to complete their self-assembly. The solvent is finally removed by dialysis or volatilization, and PEG-PLGA NPs are collected. Using this method, PEG-PLGA NPs loaded with a manganese (II) complex were prepared and they showed excellent encapsulation and drug-loading efficiency, leading to good therapeutic effect against breast cancer stem cells (Eskandari and Suntharalingam, 2019). In another study, honokiol-loaded NPs prepared by nanoprecipitation were modified to obtain nanocarriers with high-loading capacity, which enhanced anti-breast cancer activity *in vitro* and *in vivo* (Haggag et al., 2020). Nevertheless, further research is still needed to clarify how the choice of organic or aqueous phase, encapsulated drug, temperature, pH, and sequence of reagent addition influence drug loading and encapsulation into NPs (Almoustafa et al., 2017).

#### 2.2.2 Double emulsion-solvent evaporation method

In the double emulsion-solvent evaporation method, PEG, PLGA and drug are added into an organic solvent (oil phase) to prepare a water-in-oil (W/O) emulsion. The resulting emulsion is then added into a water phase, and the mixture is homogenized by sonication to obtain a W/O/W emulsion (Chen et al., 2019; Shen and TanTai, 2020). Evaporation of the organic solvent followed by filtration yields drug-loaded PEG-PLGA NPs.

Similar to the O/W single emulsion-solvent evaporation method, this approach is used to encapsulate proteins and hydrophilic drugs and limit their diffusion out of the NPs, thereby improving entrapment efficiency and sustained release (Zhang et al., 2014). For instance, this method was used to prepare salidroside-loaded PEG-PLGA NPs with high entrapment efficiency by adjusting the glycolic acid/lactic acid molar ratio and the molecular weight of PLGA. The resulting preparation showed low polydispersity index, high zeta potential, and good release and cytotoxicity properties *in vitro*, indicating that the behavior of PEG-PLGA NPs strongly depends on composition and choice of raw materials (Fang et al., 2014). Endostar-loaded PEG-PLGA NPs were also prepared by double emulsion-solvent evaporation, and they showed sustained and controlled drug release properties as well as specific tumor targeting ability *in vivo* (Hu and Zhang, 2010).

## 3 Application of drug-loaded PEG-PLGA NPs in cancer treatment

### 3.1 Chemotherapeutic applications

Although chemotherapy remains the main treatment approach for cancer, chemotherapeutic drugs suffer from low targeting ability, low cytotoxicity, fast elimination, serious side effects, and high drug resistance. PEG-PLGA NPs have emerged as a novel formulation with great biocompatibility and non-immunogenicity that can improve the solubility, stability, and safety of chemotherapeutic drugs for the treatment of various cancer types (Table 1). For example, paclitaxel (PTX)-loaded PEG-PLGA NPs rapidly prepared by microwave synthesis showed similar cytotoxicity to Taxol (Dunn et al., 2019). Satisfactory pharmacokinetic and pharmacodynamic results have also been reported for docetaxel (DTX)- and PTX-loaded PEG-PLGA NPs (Wei et al., 2013; Noori Koopaei et al., 2014). In another study, 5-fluorouracil (5-FU)-loaded PEG-PLGA NPs improved the encapsulation, controlled release, and efficacy of the drug against solid Ehrlich carcinoma, while reducing the drug’s adverse effects (Haggag et al., 2018). PEG-PLGA NPs encapsulating doxorubicin (DOX) (Nagahama et al., 2015), bendamustine (Khan et al., 2016b), Endostar (Hu and Zhang, 2010), metformin (Faramarzi et al., 2019), and manganese (II) complex (Eskandari and Suntharalingam, 2019) have shown good sustained release and anticancer properties *in vitro* and *in vivo*. In addition, PEG-PLGA NPs have been used for the co-delivery of chemotherapeutic drugs, such as 5-FU and chrysin or sorafenib and pigment epithelium-derived factor for colorectal cancer therapy, as well as gefitinib and quercetin for lung cancer treatment (Chen et al., 2019; Khaledi et al., 2020; Shen and TanTai, 2020). The co-loaded NPs demonstrated better sustained release performance, targetability, and tumor growth inhibition than single drug-loaded NPs. These results suggest that PEG-PLGA NPs can be used for the synergistic treatment of tumors, while reducing the frequency of drug administration.

**TABLE 1:** Recent applications of PEG-PLGA nanoparticles as drug carriers.

Treatment type	Preparation of drug loaded PEG-PLGA nanoparticle	Payload	Treatment model	Administration mode	Advantages	Referrence
Chemotherapy	Microwave synthesis	PTX	HeLa cell	Culture medium	Unique release and dose-dependent cytotoxicity	Dunn et al. (2019)
Chemotherapy	3-factor, 3-level Box-Behnken design	DTX	SKOV-3 cell;tumor bearing female balb/c mice	Culture medium;intravenous	Higher cytotoxic efficacy and less weight loss	Noori Koopaei et al. (2014)
Chemotherapy	Nanogel mixed system	PEGylated Taxol	4T1-luciferase cells trasplanted female balb/c mice	Intravenous	more efficient inhibition the growth	Wei et al. (2013)
Chemotherapy	Modified double emulsion method	5-FU	Solid Ehrlich carcinoma murine	Intraperitoneal injection	Reduction in tumor volume and weight, improvement on sustained release *in vitro* and anticancer efficacy *in vivo*	Haggag et al. (2018)
Chemotherapy	Ring opening melt polymerization method;double emulsion method	5-FU;Chrysin	HT29 human colon cancer cell	Culture medium	Higher growth inhibitory effects;improvement on the therapeutic and functional delivery efficacy	Khaledi et al. (2020)
Chemotherapy	Modified double-emulsion solvent evaporation	Sorafenib;PDEF	C-26 cell;HEK-293;C-26 cell trasplanted balb/c mice	Culture medium;intravenous	Higher entrapment efficiency;better sustained manner;no obvious toxicty	Chen et al. (2019)
Chemotherapy	Modified emulsification solvent evaporation	Gefitinib;quercetin	PC-9 cell;PC-9 cell trasplanted mice	Culture medium;intravenous	Higher cellular uptake and cell inhibition rates	Shen and TanTai, (2020)
Chemotherapy	Self-assembly of PLGA-PEG-PLGA copolymer micelles, CNDs, and DOX.	DOX	HeLa cell; (PC3, human prostate cancer cell line)cell trasplanted Female nude mice (BALB/cSlc-nu/nu)	Culture medium;Intratumor injection	long-term sustained antitumor activity	Nagahama et al. (2015)
Chemotherapy	Two-step surface functionalization method	Bendamustine	A549 cell;MCF-7 cell;T47D;PC-3;	Culture medium	Less hemolytic;improvement on stability and anticancer efficiency	Khan et al. (2016)
Chemotherapy	Double emulsion method	Endostar	HT-29 cell trasplanted BALB/c nude mice	Intravenous	Sustained release;improvement on anticancer activity	Hu and Zhang, (2010)
Chemotherapy	Ring-opening polymerization method	Metformin	SKOV-3 cell	Culture medium	More cytotoxicity in a time-and dose-dependentmanner;improvement on anticancer activity	Faramarzi et al. (2019)
Chemotherapy	Self-assemble in water;nanoprecipitation method	Manganese (II) complex	HMLER-shEcad cells	Culture medium	Improvement on breast cancer stem cells;reduction in toxicity	Eskandari and Suntharalingam, (2019)
Traditional Chinese medicine	Double emulsion method	Chrysin	AGS cell	Culture medium	Up regulation of expression of miR-34a;higher solubility;significant inhibitory effect in cell growth	Mohammadian et al. (2015)
Traditional Chinese medicine	Modified emulsion of oil in water	Chrysin;curcumin	SW480 cell	Culture medium	Higher bioavailability and solubility;down regulation of expression of telomerase (hTERT) gene	Bagheri et al. (2018)
Traditional Chinese medicine	Double emulsion/solvent evaporation methods	DIM;EA	Human pancreatic cancer cell line;Chick Chorioallantoic Membrane (CAM) Cancer Implant Model	Culture medium;intramodel injection	More effective suppression of pancreatic cancer cell viability, pancreatic tumor weight, implanted cancer cell viability, and tumor angiogenesis	Mousa et al. (2020)
Traditional Chinese medicine	Organic solvent volatilization method	Ginsenoside, 25-OCH3-PPD	Human prostate cancer cell lines LNCaP (p53 wild-type); DU145 (p53 mutant);PC3 (p53 null) ;human intestinal epithelial cell line Caco-2;male CD-1 mice;PC3 xenograft model	Culture medium;oral	MDM2 oncogene inhibition;steady and sustained release ;improvement on cancer cell uptake *in vitro*, tumor uptake *in vivo*, oral bioavailability, absorption, half-life and anticancer efficacy;little toxicity in mice at high doses	Voruganti et al. (2015)
Traditional Chinese medicine	Response surface (three-level) design	Icariin	ASPC-1 cell	Culture medium	Higher cytotoxicity and apoptotic potent;arrest of G2-M phase of aspc-1 cells;upregulation of caspase-3	Alhakamy, (2021)
Traditional Chinese medicine	Ring open copolymerization of lactide and glycoside;double emulsification method	Salidroside	4T1 cell ;PANC-1;SKOV-3 cell ;PC-3 cell; CT26 cell; one human normal cell line (AD293)	Culture medium	Gradually release;significant improvement *in vitro* antitumor activity of Sal in PANC-1 and 4T1 cancer cell lines;no toxicity on AD293 cells at a concentration (100 μg/ml);higher antitumor efficacy	Fang et al. (2014)

Abbreviations: DIM-3, 3′-diindolylmethane; DOX-doxorubicin;DTX-Docetaxel; EA-ellagic acid; 5-FU-5-fluorouracil; PEDF-pigment epithelium-derived factor; PEG-polyethylene glycol; PLGA-poly (lactic acid-co-glycolic acid); PTX-Paclitaxel.

### 3.2 TCM therapy

Active TCM substances have attracted increasing attention as antitumor drugs or adjuvant therapy for chemotherapy due to their multi-target ability. However, their short half-life, rapid metabolism, low bioavailability, and poor targeting ability significantly limit their application. Therefore, the encapsulation of such active substances into shells (nanoparticles, liposomes, gels, vesicles, etc.), such as PEG-PLGA NPs, can improve their properties and anticancer efficacy (Table 1). For example, chrysin-loaded PEG-PLGA NPs upregulated miR-34a and showed higher solubility and inhibitory activity than free chrysin against AGS cell growth (Mohammadian et al., 2015). PEG-PLGA NPs co-loaded with chrysin and 5-FU or curcumin also exhibited significant synergistic anticancer effects in colorectal cancer treatment (Bagheri et al., 2018; Khaledi et al., 2020). Similarly, PEG-PLGA NPs carrying both di-indolylmethane and ellagic acid effectively reduced the viability of pancreatic cancer cells and suppressed tumor growth and angiogenesis (Mousa et al., 2020). In addition, PEG-PLGA NPs loaded with ginsenosides showed better oncogene regulation, anticancer synergism, drug uptake, half-life, and safety than the corresponding free drugs (Voruganti et al., 2015). PEG-PLGA NPs co-loaded with drugs and active TCM monomers such as icariin (Alhakamy, 2021), salidroside (Fang et al., 2014), and honokiol (Haggag et al., 2020) have also shown anticancer synergistic effects and sustained release. For instance, lupeol-loaded PEG-PLGA NPs enhanced the sensitivity of hepatocellular cancer to radiotherapy (Xie et al., 2021), which may provide a new research direction for antitumor drug resistance. These results suggest that the encapsulation of existing and newly discovered active TCM substances into PEG-PLGA NPs can promote their application in cancer treatment.

### 3.3 Active targeted cancer therapy

NPs have special structure, chemical properties, and passive targeting, which allow them to encapsulate nonspecific or targeted drugs and TCM. Although NPs can enhance the targeting ability of antitumor drugs due to the ERP effect and passive enhanced permeability, they still lack the ability to target malignant cells (Tuwahatu et al., 2018; Barkat et al., 2021). To promote active targeting, the surface of PEG-PLGA NPs has been modified with various ligands, such as folate, aptamer, transferrin, and hyaluronic acid (Table 2).

**TABLE 2:** Recent applications of ligand-modified PEG-PLGA nanoparticles as drug carriers for actively targeted cancer therapy.

Modifying molecule	Modification methods	Target	Payload	Treatment model	Progressiveness compared with non-target preparation	Referrence
Folate	A three - step chemical synthesis	Folate receptors	Saquinavir	PC-3 (human prostate) cells; MCF-7 (human breast) cancer cell lines	Cell experiment:cytotoxicity; cellular uptake	Singh et al. (2015)
Folate	Carbodiimide chemistry	Folate receptors	Sorafenib	BEL7402 cells	Cell experiment:cellular uptake; suppression on cell proliferation; anticancer efficacy;inhibition on the colony forming ability	Li et al. (2015)
Folate	Covalent linkage	Folate receptors	Paclitaxel;indocyanine green;perfluorohexane	MDA-MB231 cells;tumor-bearing mice	Cell experiment :cellular uptake;anticancer effect·Transplant model experiment:accumulation in tumor tissue;targeting ability;microbubble activation;low toxicity	Liu et al. (2018)
EpCAM aptamer	Covalent linkage	Epithelial cell-adhesion molecules	Doxorubicin	A549 cell; SK-MES-1 cell;nude mice bearing SK-MES-1 non-small cell lung cancer xenografts	Cell experiment :cytotoxicity·Transplant model experiment:weight loss;toxicity; tumor inhibition	Alibolandi et al. (2015a)
EpCAM aptamer	Covalent linkage	Epithelial cell-adhesion molecules	Doxorubicin	EpCAM-positive tumor cells (MCF-7)	·Cell experiment: celluptake; internalization;cytotoxicity	Alibolandi et al. (2015b)
Transferrin	Simple amide coupling	TFR	Thymoquinone	A549 cells (TFR over-expression);chick CAM xenograft models;xenograft model in immunosuppressed Balb/c mice	·Cell experiment :nanoparticle internalization;p53 up-regulation for apoptosis·Transplant model experiment:anti-cancer activity *via* controlling the p53/miR-34a/miR-16 axis	Upadhyay et al. (2019)
Transferrin	Maleimide-thiol coupling reaction	TFR	Doxorubicin;tetrahydrocurcumin	Rat C6 glioma cell line ; human breast cancer cell line (MCF-7) ;nude mice bearing glioma xenografts	·Cell experiment :uptake;synergistic effect of radiotherapy·Transplant model experiment:drug accumulation in the brain	Zhang et al. (2019)
Biotin	Dlick reaction	Biotin receptors	Doxorubicin	4T1 cells;female Balb/C mice bearing 4T1 cell xenografts	·Transplant model experiment:improvement *in vivo* antitumor efficacy;potential of mitigating toxic	Singh et al. (2017)
Biotin	DDC/NHS chemistry method	Biotin receptors	DI	Human cervical cancer Hela cells	·Cell experiment :antiproliferative activity for preferential internalization;decreasing the intracellular reactive oxygen species (ROS) level	Luo et al. (2018)
A10 aptamer	Conjugated the RNA aptamer to the terminus of PEG-PLGA	PMSA	TFO	LNCaP cell (PMSA+);BALB/c nude mice bearing a LNCaP cell xenograft	·Cell experiment :silenced the AR gene;cytotoxicity·Transplant model experiment: cellular uptake	Jiao et al. (2016)
Hyaluronic acid	Activated carboxyl covalently linked	CD44 molecular	Cisplatin	CD44-over expressing ovarian cancer cell line (SKOV-3);Ehrlich tumor (solid) bearing mice	·Cell experiment : cytotoxicity;celluar uptake·Transplant model experiment:antitumor activity	Alam et al. (2017)
Glycyrrhetinic acid	Chemical synthesis by a two-step process	Glycyrrhetinic acid receptors	Artesunate	HepG2 cell; Hep3B cell; SMCC-7721 cell	·Cell experiment : cytotoxicity;binding affinity ;andaccumulation in hepatoma cells	Pan et al. (2020)
Chondroitin sulfate	PEG-Bis-Amine Link	Chondroitin sulfate receptors	5-fluorouracil	MCF-7/MDA-MD 231 breast cancer cells	·Cell experiment : cytotoxic effect ;hemolytic potential	Yadav et al. (2010)
Alendronate	Multistep synthesis	Mineral hydroxyapatite	Bortezomib	Female Nod/SCID beige mice injected with Luc+/GFP + MM1S cells	·Transplant model experiment:retention;accumulation; bone homing of targeted	Swami et al. (2014)
LFC131	Covalent bonding of NHS-activated PEG-PLGA nanoparticles	CXCR4	Sorafenib;metapristone (RU42633)	HCC cell lines (HepG2, Huh7, and SMMC-7721 cells);female BALB/c nude mice injected subcutaneously with human SMMC-7721 cells	·Cell experiment : ntracellular levels of drugs; anti-proliferative efficacy; tumor cell apoptosis; accumulation in tumors·Transplant model experiment:inhibitory efficacy on tumor growth	Zheng et al. (2019)
PEI	Postsynthesis of PLGA-PEG nanoparticles	SP94	TK-p53-NTR	Female nude mice (nu/nu) injected with HepG2- FLuc cell	·Transplant model experiment:gene-loaded transfer capacity ;biosafety	Li et al. (2021)
iRGD	Interaction between Mal groups of Mal-PEG-PLGA and the thiol group of iRGD for 24 h	iRGD receptors	Croconaine815	MDA-MB-231 cells;MDA-MB-231 cellbearing nude mice·Transplant model experiment:inhibition on tumor proliferation	·Cell experiment : targeting ability	Sukumar et al. (2020)
E2	Covalent conjugation	ER	Docetaxel	ER positive MCF-7 cells ;HeLa cells (ER negative);breast cancer model in female Sprague Dawley (SD) rats	·Cell experiment : cellular uptake in ER positive MCF-7 cells;cytotoxicity;·Transplant model experiment:tumor regression	Jain et al. (2015)
Pep-1(CGEMGWVRC);CGKRK(Cys-Gly-Lys-ArgLys) peptide	Emulsion/solvent evaporation method	Interleukin 13 receptor α2;heparan sulfate	Paclitaxel	Human umbilical vein endothelial cells ;rat C6 glioma cell lines;nude mice injected with C6 cells	·Cell experiment : cellular uptake;improvement of *in vitro* antiglioma activity in the respect of proliferation, tumor spheroid growth, tube formation, and migration·Transplant model experiment:targeted and accumulated at glioma site	Lv et al. (2016)

Abbreviations:DDC-dicyclohexylcarbodiimide;DI-15, 16-Dihydrotanshinone I;ER-estradiol receptors;EpCAM-epithelial cell adhesion molecular;E2-estradiol;NTR-nitroreductase;PEG-polyethylene glycol;PEI-polyethylenimine;PLGA-poly(lactic acid-co-glycolic acid);PMSA-prostate specific membrane antigen;TFO-triplex forming oligonucleotides;TFR-transferrin receptor;TK-thymidine kinase.

For example, the 5′-NH2-modified EpCAM aptamer was covalently bound on the surface of DOX-loaded PEG-PLGA NPs, and the modified NPs showed stronger inhibitory activity on tumor growth in nude mice bearing non-small cell lung cancer xenografts and higher cytotoxicity against A549 cells than unmodified NPs (Alibolandi et al., 2015a). In addition, EpCAM aptamer-conjugated PEG-PLGA NPs enhanced the cellular uptake and cytotoxicity of DOX against human breast adenocarcinoma cells (Alibolandi et al., 2015a; Alibolandi et al., 2015b). Conjugation of A10 aptamer to PEG-PLGA NPs loaded with triplex-forming oligonucleotides led to specific targeting of prostate cancer cells and inhibition of tumor growth, and the modified NPs silenced the androgen receptor gene more effectively than unmodified NPs (Jiao et al., 2016).

Transferrin has also been added to drug-loaded NPs due to its non-toxicity, biodegradability, and low expression in most normal tissues. For example, thymoquinone-loaded PEG-PLGA NPs modified with transferrin significantly induced cancer cell apoptosis by regulating the p53/miR-34a/miR-16 axis (Upadhyay et al., 2019). Multidrug-loaded PEG-PLGA NPs decorated with transferrin increased the intracerebral accumulation of the drugs and showed good anti-glioma efficacy *in vivo* (Zhang et al., 2019).

Since biotin receptors are overexpressed in cancer cells, NPs modified with biotin have been developed. Among them, 15,16-dihydrotanshinone I-loaded PEG-PLGA NPs modified with biotin prevented HeLa cell proliferation by downregulating reactive oxygen species and triggering G2/M phase cycle arrest (Luo et al., 2018). DOX-loaded PEG-PLGA NPs modified with biotin have shown good potential *in vitro* and *in vivo* for active, targeted therapy of breast cancer (Singh et al., 2017).

Hyaluronic acid can specifically bind to CD44 molecular receptors, which are involved in regulating specific cell-cell and cell-matrix interactions. Cisplatin-loaded PEG-PLGA NPs modified with hyaluronic acid were internalized to a greater extent than unmodified NPs by human ovarian cancer (SKOV-3) cells overexpressing CD44, and the modified NPs were more toxic against those cells (Alam et al., 2017).

Folate, which is highly expressed in a wide range of tumor cells, was also conjugated to PEG-PLGA NPs, affording nanomaterials with superior cytotoxicity and improved cellular uptake due to the specific binding of folate to the corresponding receptors (Singh et al., 2015). Similarly, folate-decorated PEG-PLGA NPs loaded with sorafenib showed high cellular uptake, antiproliferation activity and antitumor effects against BEL7402 cancer cells (Li et al., 2015).

PEG-PLGA NPs decorated with glycyrrhetinic acid were able to concentrate the drug artesunate in liver cancer cells (Pan et al., 2020). 5-FU-loaded PEG-PLGA NPs modified with chondroitin bound to the chondroitin sulfate receptors overexpressed on various tumor cell types, leading to higher cytotoxicity and less hemolytic effects than unmodified NPs (Yadav et al., 2010).

Targeting the tumor microenvironment has also emerged as an effective strategy for cancer treatment. For example, alendronate-loaded PEG-PLGA NPs were modified with hydroxyapatite, an abundant mineral in bone tissues with high affinity for bisphosphonates, and the formulation accumulated in bone tumors *in vivo* (Swami et al., 2014). PEG-PLGA NPs that were co-loaded with sorafenib and metapristone and were conjugated with LFC131, a peptide inhibitor of CXCR4 (Zheng et al., 2019), showed promise in bypassing CXCR4-mediated resistance of hepatocellular carcinoma tumor cells to the widely used drug sorafenib.

Recent studies have investigated the role of modified PEG-PLGA NPs in photoacoustic imaging and photothermal tumor therapy. For example, folate-conjugated PEG-PLGA NPs were co-loaded with indocyanine green, perfluorohexane, and PTX to prepare NPs that could be simultaneously used for photoacoustic and enhanced ultrasound echo imaging as well as for active targeting of tumors overexpressing folate receptors (Liu et al., 2018). PEG-PLGA NPs that were loaded with pH-sensitive croconaine815 and decorated with iRGD showed strong photoacoustic signal enhancement and effectively inhibited tumor growth, serving as a novel strategy for *in vivo* multiplexed photoacoustic imaging and pH-responsive photothermal therapy (Li S. et al., 2021). In addition, PTX-loaded PEG-PLGA NPs that were decorated with the peptides Pep-1 (CGEMGWVRC) and CGKRK (Cys-Gly-Lys-Arg-Lys) enhanced the antiglioma efficacy of PTX by inhibiting angiogenesis and killing cancer cells (Lv et al., 2016).

### 3.4 Gene-targeting cancer therapy

Cancer gene therapy uses nucleic acids, oligopeptides and proteins to treat tumors by regulating the expression of related genes. However, these drugs cannot be extensively used in clinical practice due to their fast degradation and easy elimination in the blood (Kim et al., 2016). To address these problems, polymer NPs such as PEG-PLGA NPs have been extensively studied as drug carriers due to their unique size, simple modification, good biocompatibility, stability, and low toxicity (Xin et al., 2017). For example, verapamil-modified PEG-PLGA NPs loaded with SN38 enhanced the expression of apoptosis-related genes (BAX/BCL2) and delayed drug resistance in colorectal cancer cells (Nagheh et al., 2017). PEG-PLGA NPs containing a novel peptide inhibiting the protein “Ras protein-regulator of chromosome condensation 1” may be able to inhibit breast cancer metastasis (Haggag et al., 2019). PEG-PLGA NPs co-loaded with DTX and short interfering RNA targeting the oncogene TUBB3 or co-loaded with sorafenib and metapristone targeting the SDF-1/CXCR4 axis have also shown promise against oncogenes in hepatocellular carcinoma (Zheng et al., 2019; Conte et al., 2021). PEG-PLGA NPs that were loaded with a “thymidine kinase–p53–nitroreductase” triple therapeutic gene and decorated with polyethylenimine were able to inhibit growth of hepatocellular carcinoma tumors (Sukumar et al., 2020).

## 4 Biocompatibility, toxicity, and safety of drug-loaded PEG-PLGA NPs

All formulations developed for biomedical or clinical use must be non-toxic and comply with the relevant biosafety regulations, while showing good biocompatibility, especially for intravenous or intraperitoneal injection. Although PLGA and PEG have been approved by the FDA as safe and biodegradable, few studies have explored the safety of PEG-PLGA NPs *in vivo* for further clinical application. For example, blank PEG-PLGA NPs showed negligible inhibitory effects on the growth of human colon adenocarcinoma SW-480 cells, confirming their good biocompatibility (Dimchevska et al., 2017). PEG-PLGA NPs loaded with bendamustine led to nearly 4-fold lower hemolysis than NPs without the PEG-PLGA matrix, also indicating good biocompatibility (Khan et al., 2016b). In addition, no significant cytotoxicity was observed for blank PEG-PLGA NPs toward HeLa cells (Luo et al., 2018), while the intraperitoneal injection of blank or honokiol-loaded PEG-PLGA NPs did not cause substantial damage to the liver or kidney of mice with breast cancer tumors (Haggag et al., 2020), further indicating the safety of these nanocarriers *in vivo*. Similarly, PEG-PLGA NPs loaded with the anticancer ginsenoside 25-OCH3-PPD did not cause histopathology in the liver, kidneys, lungs, spleen, heart, or brain of mice bearing PC3 xenograft tumors (Voruganti et al., 2015), while estradiol-decorated DTX-loaded PEG-PLGA NPs showed negligible cytotoxicity in MCF-7 cells and minimal hepatotoxicity in mice (Jain et al., 2015). A recent study analyzed the plasma activation levels of complement C3 induced by the intravenous injection of PEG-PLGA NPs conjugated with anti-cathepsin K antibodies. The results revealed no significant upregulation of complement C3 after treatment with the modified NPs, suggesting that they not induce an immune response (Camardo et al., 2020). DOX-loaded PEG-PLGA NPs decorated with biotin showed low hemolytic activity and cytotoxicity and proved to be safe relative to free DOX (Singh et al., 2017). Odorranalectin-modified PEG-PLGA NPs were found to be non-toxic to human lung adenocarcinoma Calu-3 cells and showed negligible toxicity and immunogenicity in the nasal cavity in toads and rats (Wen et al., 2011). PEG-PLGA NPs modified with both Pep-1 and CGKRK peptides were also shown to be non-toxic to major organs in mice (Lv et al., 2016).

Nevertheless, the influence of different formulation ratios, molecular weights, and synthesis methods on the safety of PEG-PLGA NPs has not been adequately explored. It is important to analyze the relationship between the toxicity of NP carriers and their physical characteristics, including polydispersity index, zeta potential, entrapment efficiency, and morphology. In fact, the entire process from raw materials to synthesis and modification of drug-loaded PEG-PLGA NPs should be rigorously optimized to maximize safety before clinical trials. Ultimately, uniform guidelines for synthesizing and formulating PEG-PLGA NPs are needed in order to ensure their efficacy as drug carriers for targeted cancer treatment.

## 5 Conclusion and prospects

The rapid growth of nanotechnology has led to the emergence of many novel therapeutic methods such as nanodrug delivery systems. The present review shows that amphiphilic block copolymer PEG-PLGA NPs can be safely used as drug nanocarriers that show sustained release properties as well as improved drug bioavailability and stability *in vivo*. PEG-PLGA NPs modified with ligands can target specific receptors on the tumor surface, enhancing tumor targeting. However, the biocompatibility, toxicity, and safety of these nanocarriers require further research to guarantee their clinical application. New cancer-specific target molecules are constantly being discovered, and studies should continue to explore how to modify NPs in order to recognize tumors. This approach may create new possibilities for precision anti-cancer treatment and diagnostic imaging.
